# Teaching pediatric otoscopy skills to the medical student in the clinical setting: preceptor perspectives and practice

**DOI:** 10.1186/s12909-020-02307-x

**Published:** 2020-11-16

**Authors:** Caroline R. Paul, Alanna D. Higgins Joyce, Gary L. Beck Dallaghan, Meg G. Keeley, Corinne Lehmann, Suzanne M. Schmidt, Kari A. Simonsen, Cynthia Christy

**Affiliations:** 1 Department of Pediatrics, University of Wisconsin School of Medicine and Public Health, 2870 University Avenue, Suite 200, Madison, WI 53705 USA; 2 Department of Pediatrics, Northwestern University Feinberg School of Medicine, Chicago, IL USA; 3 University of North Carolina School of Medicine, Office of Medical Education, Chapel Hill, North Carolina USA; 4 Department of Pediatrics, University of Virginia School of Medicine, Charlottesville, Virginia USA; 5 Department of Pediatrics, University of Cincinnati College of Medicine, Cincinnati, OH USA; 6 Department of Pediatrics, University of Nebraska Medical Center, Omaha, NE USA; 7 Department of Pediatrics, University of Rochester School of Medicine and Dentistry, Rochester, New York USA

**Keywords:** Pediatric otoscopy, Ear exam, Acute otitis media, Faculty development, Clinical educator, Clinical teaching, Core clinical skills

## Abstract

**Background:**

Acute otitis media (AOM) is the most frequent indication for antibiotic treatment of children in the United States. Its diagnosis relies on visualization of the tympanic membrane, a clinical skill acquired through a deliberate approach. Instruction in pediatric otoscopy begins in medical school. Medical students receive their primary experience with pediatric otoscopy during the required pediatric clerkship, traditionally relying on an immersion, apprentice-type learning model. A better understanding of their preceptors’ clinical and teaching practices could lead to improved skill acquisition. This study investigates how pediatric preceptors (PP) and members of the Council on Medical Student Education in Pediatrics (COMSEP) perceive teaching otoscopy.

**Methods:**

A 30-item online survey was administered to a purposeful sample of PP at six institutions in 2017. A comparable 23-item survey was administered to members through the 2018 COMSEP Annual Survey. Only COMSEP members who identified themselves as teaching otoscopy to medical students were asked to complete the otoscopy-related questions on the survey.

**Results:**

Survey respondents included 58% of PP (180/310) and 44% (152/348) of COMSEP members. Forty-one percent (62/152) of COMSEP member respondents identified themselves as teaching otoscopy and completed the otoscopy-related questions. The majority agreed that standardized curricula are needed (PP 78%, COMSEP members 97%) and that all graduating medical students should be able to perform pediatric otoscopy (PP 95%, COMSEP members 79%). Most respondents reported usefulness of the American Academy of Pediatrics (AAP) AOM guidelines (PP 95%, COMSEP members 100%). More COMSEP members than PP adhered to the AAP’s diagnostic criteria (pediatric preceptors 42%, COMSEP members 93%). The most common barriers to teaching otoscopy were a lack of assistive technology (PP 77%, COMSEP members 56%), presence of cerumen (PP 58%, COMSEP members 60%), time to teach in direct patient care (PP 46%, COMSEP members 48%), and parent anxiety (PP 62%, COMSEP members 54%).

**Conclusions:**

Our study identified systemic and individual practice patterns and barriers to teaching pediatric otoscopy. These results can inform education leaders in supporting and enabling preceptors in their clinical teaching. This approach can be adapted to ensure graduating medical students obtain intended core clinical skills.

## Background

Acute otitis media (AOM) is the most frequent indication for antibiotic treatment of children in the United States. Approximately 40–50% of children will have more than one episode of AOM before the age of 2 years [1, 2]. It is estimated that over 5 million cases of AOM occur annually in United States children, resulting in more than 10 million prescriptions for antibiotics [1]. The threat of rising healthcare costs, growing antibiotic resistance, and increased surgical referrals for recurrent AOM combine to make the accurate diagnosis of AOM important [1, 3–5].

Competency in the pediatric ear exam is critical to the accurate diagnosis and appropriate management of pediatric ear disease [1, 3]. The American Academy of Pediatrics (AAP) 2013 Clinical Practice Guideline stresses that diagnosis of AOM relies on adequate visualization of the tympanic membrane [1], a clinical skill acquired through a deliberate, stepwise approach [4, 6].

The AAP recommends that instruction in the evaluation of the middle ear begin “in medical school and continue throughout postgraduate training.” [1] Especially given that physicians other than pediatricians will diagnose children with AOM, delivering standardized otoscopy curricula to trainees in medical school is “an absolute necessity” for “the most widespread effect” on clinical practice [7]. Despite expert recommendations, peer-reviewed curricula in pediatric otoscopy have emerged only recently [4, 6, 8, 9]. Such curricula have demonstrated gains in knowledge and skills that translate to the clinical setting [4, 6]. However, their actual use in clinical teaching is unknown and relies on the individual faculty responsible for teaching pediatric otoscopy. In addition to peer-reviewed curricula, advances in technology, such as digital video otoscopes to improve visualization of the tympanic membrane, have emerged [5, 10, 11]. Mannequins have also been developed to effectively teach and assess pneumatic otoscopy skills [6, 8]. However, standardized use and evaluation of these tools in the clinical teaching setting are still lacking.

Medical students receive their main clinical exposure to pediatric otoscopy during the required third-year pediatric clerkship, traditionally relying on an immersion, apprentice-type learning model in primary care ambulatory settings. The Council on Medical Student Education in Pediatrics (COMSEP) clerkship curriculum recommends that medical students should be able to “observe the tympanic membrane using an otoscope and an insufflator” [12, 13]. A clear standard teaching practice for otoscopy is not evident in the literature; anecdotally, current practice is a mixture of variable didactics and apprentice-type teaching.

One needs assessment revealed that student expectations of developing expertise in pediatric otoscopy were not met by the end of their pediatric clerkship. In addition, students reported anxiety with the pediatric ear exam even after completing the pediatric clerkship. Critical learning opportunities were missed, with students reporting inadequate observation of their exam technique and few supervising physicians providing feedback on students’ skills [4].

Given the critical teaching role of pediatric preceptors, it is important to understand preceptor practice and teaching patterns and their use of existing curricula and AAP Guidelines in teaching otoscopy. Learners’ perceptions of *their* preceptors’ teaching practice have been reported [4, 14]. Yet, little is known about preceptors’ own otoscopy skills, their teaching needs, or their perceptions and attitudes toward teaching pediatric otoscopy to medical students. Van Uum explored general practitioners’ views and expectations regarding pain management of AOM. But we are not aware of any studies regarding preceptors’ and other clinicians’ teaching and clinical behaviors and attitudes in this area [15]. A greater understanding of preceptor clinical and teaching reported practices and attitudes regarding pediatric otoscopy education could lead to improved skill acquisition and impact patient outcomes.

Thus, we sought to inquire about preceptors’ perceptions and attitudes about both their clinical and teaching practices. “The best use of survey methodology is to investigate human phenomena … that are neither directly observable, nor available in documents.” [16] Thus, we chose survey methodology to acquire this information with the use of a novel survey instrument that focused on our key domains of inquiry. We aimed to investigate how pediatric preceptors (PP) and members of the Council on Medical Student Education in Pediatrics (CM) perceive their own clinical skills and the teaching of otoscopy to medical students during the pediatric clerkship, including barriers to teaching in the outpatient clinical setting.

## Methods

In 2017, pediatric educators from six different academic institutions developed a survey to send to pediatric ambulatory clinical preceptors affiliated with their home institutions. All participating institutions were academic teaching hospitals affiliated with a medical school whose pediatric clerkships contained outpatient primary care rotations. The pediatric preceptors were considered for inclusion because they supervised medical students in the ambulatory setting. Settings included ambulatory academic pediatric teaching clinics, private practices, and federally funded clinics. Recruitment criteria and identification of eligible preceptors were determined by group consensus by the research team. Each site obtained ethics approval to conduct the Preceptor Survey, and the lead author obtained ethics approval for questions included in the COMSEP Survey.

In 2018, questions were submitted for inclusion in the 2018 COMSEP Annual Survey. This survey is sent to every member of COMSEP and is a venue for members to undertake survey research. To keep the survey manageable, the COMSEP Survey Committee reviews submissions and determines which studies will be included in the annual survey. The survey was one of four chosen to be included in the 2018 COMSEP Annual Survey. With the branching logic embedded in the survey, the survey asked participants to self-select if they teach and/or oversee the teaching of otoscopy skills to medical students on the required pediatrics clerkship. The COMSEP Survey respondents who met this criteria were asked to complete the otoscopy-related questions.

The design of the Preceptor Survey and COMSEP Survey followed survey design practices outlined by Artino and colleagues [17]. Peer-reviewed standards informed the content of both surveys [1, 6]. The final surveys were created through an iterative process. First, expert group consensus generated general themes of inquiry for a focus group. A focus group, consisting of 10 ambulatory general pediatricians at one site, further explored the content domains of the surveys. Findings from the focus group confirmed the domains of inquiry (i.e. choices for barriers to teaching, use of technology) for the surveys and further refined the question items. A survey design expert reviewed the surveys with subsequent revisions. Finally, the surveys were further refined through a pilot evaluation on three general practicing pediatricians at the lead author’s institution, with input from two additional pediatric educators. The results of the pilot survey were not included in the overall data analysis. The pilot evaluation did not alter the content of the questions. However, wording of some questions was edited for greater clarity to obtain more accurate responses.

The Preceptor Survey focused on the following domains: attitudes about pediatric otoscopy, medical student teaching, teaching barriers, and preceptors’ clinical practices. The COMSEP Survey included similar domains, and in addition, explored COMSEP members’ views about their faculty who taught pediatric otoscopy. The COMSEP Survey excluded questions contained in the Preceptor Survey that queried the demographics of preceptors and personal clinical practice patterns. The majority of questions for the COMSEP Survey were derived from the Preceptor Survey. Additional questions were added in order to examine the perceptions of education leaders about their faculty who taught pediatric otoscopy and AOM to medical students. In addition to the above mentioned developmental process, the COMSEP Survey underwent an additional pilot evaluation at two investigators’ sites with subsequent revision.

**Fig. 1:**
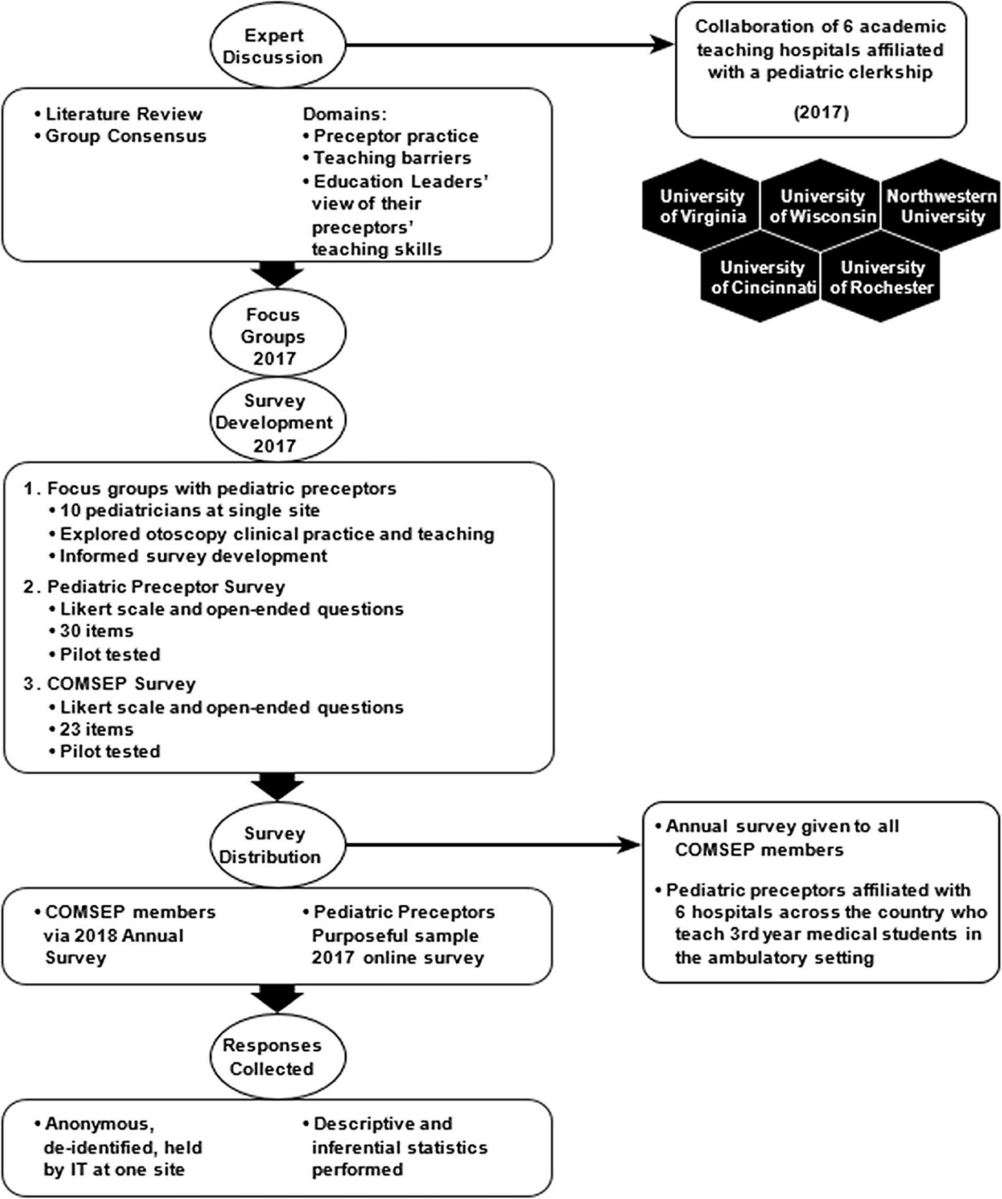
Development and implementation of surveys

Both surveys were administered by directly emailing the targeted subjects, with a link to the survey included in the email message. The preceptor survey was built in the Qualtrics online survey platform (Qualtrics XM, Provo, Utah) at the lead author’s institution. Each site was provided a link for the survey to send to their preceptors. For the COMSEP Survey, the organization’s survey committee administered the survey to all of its members. Study investigators received de-identified, anonymous data for both the Preceptor and COMSEP Surveys, as well as demographic data for respondents of the COMSEP Survey (Fig. 1). The Preceptor Survey was implemented at the different sites from September 15, 2017 to October 31, 2017. The COMSEP Survey was implemented from March 28, 2018 to May 15, 2018.

As described previously, content validity for the surveys was achieved through literature reviews, expert opinion, and focus groups with other experts. Test-retest reliability comparing preceptors with COMSEP members resulted in a Cronbach’s alpha of 0.85.

G*Power (Universität Kiel, Germany) was used to calculate a minimum sample size. For comparisons across institutions with a Wilcoxon signed-rank test, an effect size of .5, Alpha of .05, and power of .95 indicated the minimum sample size needed was 57 participants.

Descriptive and inferential statistics performed with SPSS software version 25 (IBM, Chicago, Illinois) were used to analyze the results of the Preceptor and COMSEP Surveys. Mann-Whitney U Test were used to compare PP and CM responses to specific questions on the surveys. Statistical significance was defined as a *p* < 0.05.

## Results

The response rate for the Preceptor Survey was 58% (181/310). The overall response rate for the 2018 COMSEP Annual Survey was 44% (152/348). Forty-one percent (62/152) of the respondents of the survey self-identified themselves as teaching and/or overseeing the teaching of otoscopy skills to medical students and answered the otoscopy-related questions.

### Medical student education

Ninety-five percent of PP and 79% of CM reported that all graduating medical students should be able to perform pediatric otoscopy, defined as visualization of the tympanic membrane using an otoscope. Additionally, 78% of PP and 97% of CM reported that a standardized curriculum for teaching pediatric otoscopy skills to medical students would enable preceptors to be more effective in their teaching.

### Preceptor clinical skills

Ninety-five percent of PP and 100% of CM reported that the AAP 2013 Clinical Practice Guideline was useful for the diagnosis of AOM. However, when asked what specific diagnostic criteria they use to make the diagnosis of AOM in clinical practice, only 42% of PP correctly selected the recommended criteria of the AAP Guidelines, which is moderate to severe bulging of the tympanic membrane.

Fifty-eight percent of PP and 47% of CM reported that skill in pneumatic otoscopy was important to the diagnosis of AOM. Yet, only 15% of PP and 35% of CM reported utilizing pneumatic otoscopy to diagnose AOM.

Forty-four percent of PP and 23% of CM reported receiving no formal training in cerumen removal. Eighty-three percent of PP and 40% of CM reported demonstrating cerumen removal to students. Thirty-seven percent of PP and 29% of CM reported difficulty in removing cerumen.

### Barriers to teaching

The most commonly reported barriers to teaching otoscopy skills were a lack of technological devices such as video otoscopes, tympanograms, and dual head otoscopes for teaching (PP 77%, CM 56%). Participants noted additional barriers to teaching including the presence of cerumen (PP 58%, CM 60%), time to teach in direct patient care (PP 46%, CM 48%), and parent anxiety (PP 62%, CM 54%). Some respondents in both groups also reported their personal teaching skills (23% PP, 21% CM) and pneumatic otoscopy skills (40% PP, 24% CM) as barriers to teaching.

**Fig. 2:**
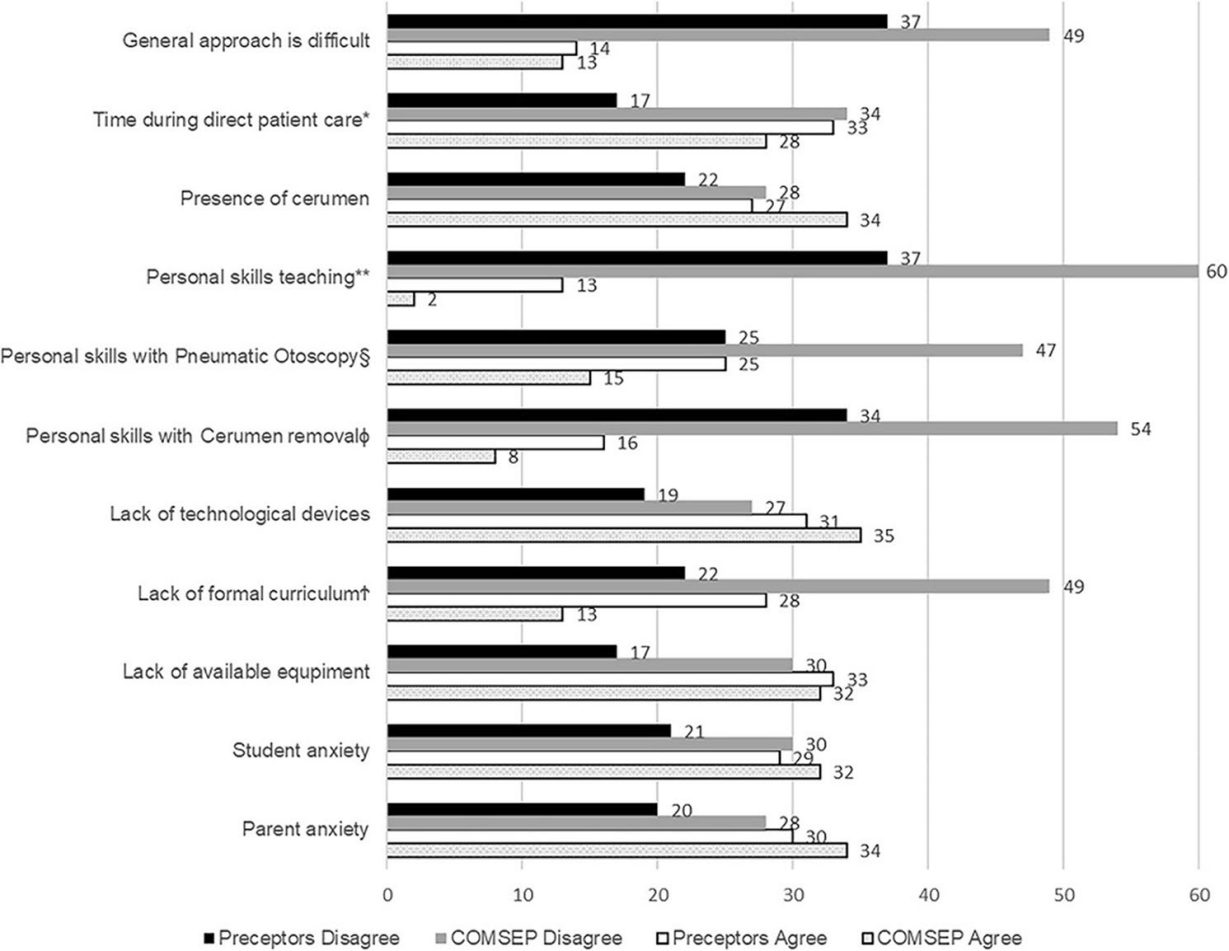
PP barriers significantly different than CM (significance (1-tailed), *p* < .05). Frequency of respondents indicated on the graph. * time during direct patient care (U = 1138.5, *p* = .005). ** personal teaching skills (U = 761.0, *p* = .001). § pneumatic otoscopy skills (U = 998.0, *p* = .001). ɸ cerumen removal skills (U = 923.0, *p* = .001). Ϯ no formal curriculum (U = 1010.0, *p* = .001)

There were differences between the PP and CM responses regarding barriers to teaching otoscopy skills. PP reported more than CM that time to teach during direct patient care (mean rank PP = 64.73 vs. CM = 49.86, *p* = .005) and personal teaching skills (mean rank PP = 72.28 vs. CM = 43.77, *p* = .001) were barriers. PP also reported more than CM that their personal skill in pneumatic otoscopy (mean rank PP = 67.54 vs. CM = 47.60, *p* = .001) and cerumen removal (mean rank PP = 69.04 vs. 46.39, *p* = .001) were barriers. PP also reported more than CM that a lack of formal curriculum (mean rank PP = 67.30 vs. CM 47.79, *p* = .001) was a barrier (Fig. 2).

## Discussion

Most medical students receive the majority of their training in the pediatric ear exam during the ambulatory component of the required pediatrics clerkship, where preceptors serve as their “front line” teachers. Proper pediatric otoscopy is essential to the accurate diagnosis of AOM. Despite this emphasis in the most recent AAP 2013 Clinical Practice Guidelines on AOM, literature on the use of the AAP Guidelines, implementation of standardized curricula, and teaching in real-time clinical settings are lacking [1, 6, 8]. Little is known about the informal training that preceptors deliver to their learners on pediatric otoscopy, although it is clear that preceptor practices and attitudes can affect student performance of these skills [9]. Gaining an understanding of preceptors’ clinical and teaching practices is a crucial first step toward improving student learning and skill acquisition, which can ultimately impact patient outcomes [1, 6, 8].

Preceptors (PP) and education leaders (CM) in our study strongly agreed that all graduating medical students should learn basic pediatric otoscopy skills and that there is a need for the implementation of standardized curricula for effective teaching. But, preceptors need practical strategies, support, and infrastructure in their clinics to implement these curricula. Future work should focus on incorporating curricula to the teaching of pediatric otoscopy skills in the clinical setting, with direct evaluation and feedback on the learned skills. For instance, the otolaryngology education literature demonstrates many efforts aimed to help otolaryngology specialists teach their learners with varying modalities [18, 19]. Similarly, faculty development for the primary care preceptor should be offered, to help such educators teach standardized content in real patient care settings.

Preceptors and education leaders cited similar multi-faceted barriers to teaching pediatric otoscopy. Some reported barriers were expected, such as the time required to teach effectively in direct patient care settings and lack of clinic supplies. Barriers related to psychosocial concerns such as student and/or parent anxiety were also anticipated results. However, difficulty with procedural skills such as pneumatic otoscopy and cerumen removal may be the most surprising reported barrier. If preceptors feel that they lack these skills, it makes sense that they would be hesitant to teach the same skill to students. Indeed, such reported difficulties may influence basic otoscopy teaching as the presence of cerumen can influence the diagnostic accuracy of AOM [1]. Of note, the presence of cerumen as a barrier has been described in other specialties as well [20].

Furthermore, emerging device technology may play a future role in faculty development in teaching otoscopy skills and lead to improved diagnostic accuracy, appropriate management, and better patient care. Whether to aid with tympanic membrane visualization using image capture of the tympanic membrane or for skills-training with simulation, advances should be explored in the context of improving diagnostic accuracy in the clinical settings [21, 22]. However, it should be noted that under resourced clinics may find it challenging to implement such emerging technology. Such technology should be developed also in the context of primary care clinical practices which provide teaching in direct patient care settings.

Our study aimed mainly to investigate preceptors’ teaching practices. Yet, the results also revealed interesting self-reports about preceptors’ own knowledge and skills. An advantage of our chosen study methodology is that a survey can reveal undocumented non-observed human phenoma [16]. These findings are important with a clinical skill such as pediatric otoscopy, where preceptor modelling remains the key learning strategy in direct patient care settings. The educational experiences of our learners may reflect the preceptors’ strengths, deficiencies, and variability. Our findings suggest some discrepancy between preceptors’ acknowledgement of the importance of the AAP Guideline and their reported clinical practice. For instance, the majority of preceptors did not choose correctly, the main diagnostic criteria of the AAP Guidelines as the criteria they use in their *own* practice. These self-reported skill deficiencies and discrepancies between AAP Guideline and actual clinical practice influence the learning of students and residents in the clinical setting and reveal the need for continued faculty development, even for the experienced and academically-oriented preceptor. Opportunities for such knowledge and skill development for pediatric preceptors and other faculty could be offered at national and regional AAP educational meetings and also be considered at other specialty meetings such as Family Medicine [20].

Some pediatric preceptors reported teaching and demonstrating skills such as pneumatic otoscopy and cerumen removal that were not taught to them and that they themselves still find difficult to perform. Many of the preceptors and educators were not performing pneumatic otoscopy, even though they selected pneumatic otoscopy as the ideal method for diagnosis. Furthermore, a large proportion of preceptors and educators who identified cerumen as a key barrier still themselves reported deficiencies with cerumen removal. Our study reveals preceptors’ barriers and deficiencies in their own practices that might not have otherwise been identified. Our results suggest that faculty competency in specific clinical skills that they are responsible for teaching cannot always be assumed. This also can be seen as an opportunity for improvement, with targeted teaching interventions for the preceptors themselves. As with other clinical skills, skill demonstration with real patients is an important teaching strategy.

The reported variance of our participants from standard guidelines is not unique. Despite clear expert recommendations, clinicians’ otoscopy practice patterns continue to vary widely, often deviating from the AAP Guideline. Other studies have also found that many providers do not routinely perform pneumatic otoscopy, an AAP recommended component in the pediatric ear exam. In addition, it is unclear if the AAP guidelines have impacted the practice of pediatric otoscopy with regards to cerumen removal. Cerumen, which is present in most pediatric patients, can complicate tympanic membrane visualization. Marchisio suggests some pediatrician reluctance to remove cerumen when the final diagnosis is AOM. Our findings echo the findings from Marchiso and Shah-Becker [23, 24]. Despite the AAP Guidelines stating cerumen as a barrier in diagnostic accuracy, 37% of preceptors and 23% of education leaders in our study who identified cerumen as a key barrier still themselves reported deficiencies with cerumen removal.

While this study focused on a core pediatric skill, the survey methodology can be used to identify how other core clinical skills are currently being taught [7]. Formal and deliberate efforts are needed to ensure that graduating students are truly equipped with the skill sets presumed to have been learned in the clinical setting. We anticipate our findings to help inform curriculum development, learning strategies, and faculty development for those preceptors responsible for teaching these skill sets in clinical settings.

Our study has limitations. Although our study surveyed a range of preceptors and educational leaders in pediatrics, it did not include family medicine and emergency medicine physicians who may also teach pediatric otoscopy to medical students. In addition, although the surveys focused on faculty self-reported skills and knowledge, it did not examine actual faculty competency in both skills and teaching. Some of our preceptors acknowledged a lack of specific skills training during their residency and reported current difficulty with the same skills that they are responsible for teaching. Our study also made some inferences based on comparison of faculty’s responses to recognized standard of care versus their reported actual practice.

## Conclusions

Pediatric otoscopy has been an assumed competency with vague and variable benchmarks for different levels of learners including the graduating medical student. Differences in competency may also exist among current faculty by their own report [1, 3]. Better dissemination and implementation of the AAP Guidelines on AOM for all learner groups, including faculty, may lead to better diagnosis of AOM and improved outcomes for patients. Our findings suggest that more global attention to skills and knowledge of preceptors who teach on the “front lines” would be of value. Our study suggests that faculty competency and skills in performing and teaching otoscopy in the direct patient care settings is a crucial first step.

## Supplementary information

**Additional file 1.** 2018 COMSEP Annual Survey.

**Additional file 2.** Preceptor Survey.

## Data Availability

Data beyond what is included in the Results and Figures can be made available after deidentification processes have occurred with both survey platforms.

## Abbreviations

AOM: Acute otitis media
AAP: American Academy of Pediatrics
COMSEP: Council on Medical Student Education in Pediatrics
PP: Pediatric preceptor
CM: Member of Council on Medical Student Education in Pediatrics

## References

[1] Lieberthal AS, Carroll AE, Chonmaitree T (2013). The diagnosis and management of acute otitis media. Pediatrics..

[2] Wald ER (2017). Acute Otitis Media 2017.

[3] Coker TR, Chan LS, Newberry SJ (2010). Diagnosis, microbial epidemiology, and antibiotic treatment of acute otitis media in children: a systematic review. JAMA..

[4] Paul CR, Gjerde CL, McIntosh G, Weber LS (2017). Teaching the pediatric ear exam and diagnosis of acute otitis media: a teaching and assessment model in three groups. BMC Med Educ..

[5] Silverston P (2016). The firefly digital otoscope as an aid to teaching otoscopy in primary care. Educ Prim Care..

[6] Paul CR, Keeley MG, Rebella GS, Frohna JG (2018). Teaching pediatric otoscopy skills to pediatric and emergency medicine residents: a cross-institutional study. Acad Pediatr..

[7] Varrasso DA (2006). Otitis media: the need for a new paradigm in medical education. Pediatrics..

[8] Paul CR, Keeley MG, Rebella G, Frohna JG (2016). Standardized checklist for otoscopy performance evaluation: a validation study of a tool to assess pediatric otoscopy skills. MedEdPORTAL..

[9] Higgins Joyce A, Raman M, Beaumont JL, Heiman H, Adler M, Schmidt SM (2019). A survey comparison of educational interventions for teaching pneumatic otoscopy to medical students. BMC Med Educ..

[10] Mousseau S, Lapointe A, Gravel J (2018). Diagnosing acute otitis media using a smartphone otoscope; a randomized controlled trial. Am J Emerg Med..

[11] Shah MU, Sohal M, Valdez TA, Grindle CR (2018). iPhone otoscopes: currently available, but reliable for tele-otoscopy in the hands of parents?. Int J Pediatr Otorhinolaryngol..

[12] Curriculum Competencies and Objectives: Skills. https://www.comsep.org/curriculum-competencies-and-objectives/. Accessed 02/06/20, 2020.

[13] COMSEP Survey. https://www.comsep.org/annual-survey/. Accessed 02/06/20, 2020.

[14] Niermeyer WL, Philips RHW, Essig GF, Moberly AC (2019). Diagnostic accuracy and confidence for otoscopy: are medical students receiving sufficient training?. Laryngoscope..

[15] van Uum RT, Sjoukes A, Venekamp RP (2018). Pain management in acute otitis media: a qualitative study exploring GPs' views and expectations parallel to a trial of an educational intervention. BJGP Open..

[16] Phillips AW (2017). Proper applications for surveys as a study methodology. West J Emerg Med..

[17] Artino AR, Durning SJ, Sklar DP (2018). Guidelines for reporting survey-based research submitted to academic medicine. Acad Med..

[18] Davies J, Djelic L, Campisi P, Forte V, Chiodo A (2014). Otoscopy simulation training in a classroom setting: a novel approach to teaching otoscopy to medical students. Laryngoscope..

[19] Wu V, Sattar J, Cheon S, Beyea JA (2018). Ear disease knowledge and otoscopy skills transfer to real patients: a randomized controlled trial. J Surg Educ..

[20] Hauk L (2017). Cerumen impaction: an updated guideline from the AAO-HNSF. Am Fam Physician..

[21] Wickens B, Lewis J, Morris DP, Husein M, Ladak HM, Agrawal SK (2015). Face and content validity of a novel, web-based otoscopy simulator for medical education. J Otolaryngol Head Neck Surg..

[22] Hakimi AA, Lalehzarian AS, Lalehzarian SP, Azhdam AM, Nedjat-Haiem S, Boodaie BD (2019). Utility of a smartphone-enabled otoscope in the instruction of otoscopy and middle ear anatomy. Eur Arch Otorhinolaryngol..

[23] Shah-Becker S, Carr MM (2018). Current management and referral patterns of pediatricians for acute otitis media. Int J Pediatr Otorhinolaryngol..

[24] Marchisio P, Pipolo C, Landi M (2016). Cerumen: a fundamental but neglected problem by pediatricians. Int J Pediatr Otorhinolaryngol..

