# Supplementary material for: Teaching pediatric otoscopy skills to the medical student in the clinical setting: preceptor perspectives and practice
Source: BMC Med Educ. 2020 Nov 16;20:429. doi: 10.1186/s12909-020-02307-x (PMC7667741; doi:10.1186/s12909-020-02307-x)
Supplement: Supplementary file 1 — Additional file 1. 2018 COMSEP Annual Survey. [file 12909_2020_2307_MOESM1_ESM.docx]

**2018 COMSEP Annual Survey**

If you are a preceptor that teaches students in the ambulatory setting, please answer the following questions.

1. **How do you teach pediatric otoscopy to medical students? Check all that apply.**

- Formal didactic session
- Formal hands-on and/or simulation session
- Direct patient care settings

1. **Do you demonstrate pneumatic otoscopy?**

- Yes
- No

1. **Do you demonstrate cerumen removal?**

- Yes
- No

1. **Please indicate you level of agreement with the following statement. I find the following barriers to teaching pediatric otoscopy to medical students:**

|  | Strongly  Agree | Agree | Disagree | Strongly  Disagree |
| --- | --- | --- | --- | --- |
| The general approach (i.e. holding young child, equipment, otoscope technique) is difficult to teach |  |  |  |  |
| The time it takes to teach students in direct patient care settings |  |  |  |  |
| The presence of cerumen |  |  |  |  |
| My own skills in teaching pediatric otoscopy |  |  |  |  |
| My own skills in performing pneumatic otoscopy |  |  |  |  |
| My own skills in cerumen removal |  |  |  |  |
| Lack of technological devices to facilitate teaching (e.g. video otoscope, dual head otoscope, tympanogram) |  |  |  |  |
| Lack of formal feasible curricula |  |  |  |  |
| Availability of equipment (e.g. insufflator bulb) |  |  |  |  |
| Student anxiety (10) |  |  |  |  |
| Parent anxiety (11) |  |  |  |  |

1. **It is important for *all* graduating medical students to know how to perform basic pediatric otoscopy, defined as visualization of the tympanic membrane using an otoscope.**

- Strongly agree
- Agree
- Disagree
- Strongly disagree

1. **A curriculum regarding how to teach the pediatric ear exam to medical students in direct patient care settings would help me to be more effective in teaching this skill.**

- Strongly agree
- Agree
- Disagree
- Strongly disagree

The next set of questions relate to your own clinical experience with pediatric otoscopy.

1. **Which criteria do you use to diagnose the majority of your patients with acute otitis media? Check all that apply.**

- Moderate to severe bulging of the TM
- Distinct erythema and poor mobility of the TM
- Poor mobility and fluid levels of the TM
- Retraction and abnormal color of the TM
- Poor mobility of the TM

1. **The AAP Guidelines for the diagnosis of Acute Otitis Media are helpful to me.**

- Strongly agree
- Agree
- Disagree
- Strongly disagree

1. **In which clinical situations do you use pneumatic otoscopy? Check all that apply.**

- To diagnose AOM
- To diagnose OME
- Only when I am unsure of the diagnosis
- I do not use insufflation

1. **Cerumen is a frequent barrier to the accurate diagnosis of Acute Otitis Media.**

- Strongly agree
- Agree
- Disagree
- Strongly disagree

1. **I find it difficult to remove cerumen in the young child.**

- Strongly agree
- Agree
- Disagree
- Strongly disagree

1. **Were you taught how to perform cerumen removal on a young child?**

- Yes
- No

1. **Were you taught how to perform pneumatic otoscopy on a young child?**

- Yes
- No

1. **Skill in pneumatic otoscopy is important to the diagnosis of AOM**

- Strongly agree
- Agree
- Disagree
- Strongly disagree

These questions relate to you as a physician:

1. **How many years have you practiced ambulatory pediatrics (post-residency)?**

- 0-5
- 5-15
- 15-25
- 25+

1. **How would you define your residency-training program?**

- Academic/Tertiary Care
- Community Pediatrics

If you are in a supervisory role for the pediatric clerkship, please answer the following questions.

1. **The preceptors in my program demonstrate pneumatic otoscopy to our clerkship students.**

- Strongly agree
- Agree
- Disagree
- Strongly disagree

1. **The preceptors in my program demonstrate cerumen removal to our clerkship students.**

- Strongly agree
- Agree
- Disagree
- Strongly disagree

1. **Please indicate your level of agreement with the following statement. The following factors are barriers for the preceptors in my program teaching pediatric otoscopy to medical students:**

|  | Strongly  Agree | Agree | Disagree | Strongly  Disagree |
| --- | --- | --- | --- | --- |
| The general approach (i.e. holding young child, equipment, otoscope technique) is difficult to teach |  |  |  |  |
| The time it takes to teach students in direct patient care settings |  |  |  |  |
| The presence of cerumen |  |  |  |  |
| My own skills in teaching pediatric otoscopy |  |  |  |  |
| My own skills in performing pneumatic otoscopy |  |  |  |  |
| My own skills in cerumen removal |  |  |  |  |
| Lack of technological devices to facilitate teaching (e.g. video otoscope, dual head otoscope, tympanogram) |  |  |  |  |
| Lack of formal feasible curricula |  |  |  |  |
| Availability of equipment (e.g. insufflator bulb) |  |  |  |  |
| Student anxiety (10) |  |  |  |  |
| Parent anxiety (11) |  |  |  |  |

1. **Are insufflator bulbs routinely available where your faculty teaches students?**

- Yes
- No
- I don’t know

1. **The preceptors in my program need further education to be more effective teachers of pediatric otoscopy to our clerkship students.**

- Strongly agree
- Agree
- Disagree
- Strongly disagree

1. **Please** **provide any other comments on the practice or the teaching of pediatric otoscopy.**

____________________________________________________________________________________________________________________________________________________________________________________________________________________________________________________________________________________________________
