# Supplementary material for: Teaching pediatric otoscopy skills to the medical student in the clinical setting: preceptor perspectives and practice
Source: BMC Med Educ. 2020 Nov 16;20:429. doi: 10.1186/s12909-020-02307-x (PMC7667741; doi:10.1186/s12909-020-02307-x)
Supplement: Supplementary file 2 — Additional file 2. Preceptor Survey. [file 12909_2020_2307_MOESM2_ESM.docx]

**2017 Pediatric Ambulatory Preceptor Survey**

The following questions relate to how you teach pediatric otoscopy to third-year medical students in ambulatory settings.

1. **How do you teach pediatric otoscopy to medical students? Check all that apply.**

- Formal didactic session (1)
- Formal hands-on and/or simulation session (2)
- Direct patient care settings (3)

1. **Do you demonstrate pneumatic otoscopy?**

- Yes (1)
- No (2)

1. **Do you demonstrate cerumen removal?**

- Yes (1)
- No (2)

1. **Please indicate your level of agreement with the following statement. I find the following factors are barriers to teaching Pediatric Otoscopy to medical students:**

|  | Strongly  Agree (1) | Agree (2) | Disagree (3) | Strongly  Disagree (4) |
| --- | --- | --- | --- | --- |
| The general approach (i.e. holding young child, equipment, otoscope technique) is difficult to teach (1) |  |  |  |  |
| The time it takes to teach students in direct patient care settings (2) |  |  |  |  |
| The presence of cerumen (3) |  |  |  |  |
| My own skills in teaching pediatric otoscopy (4) |  |  |  |  |
| My own skills in performing pneumatic otoscopy (5) |  |  |  |  |
| My own skills in cerumen removal (6) |  |  |  |  |
| Lack of technological devices to facilitate teaching (e.g. video otoscope, dual head otoscope, tympanogram) (7) |  |  |  |  |
| Lack of formal feasible curricula (8) |  |  |  |  |
| Availability of equipment (e.g. insufflator bulb) (9) |  |  |  |  |
| Student anxiety (10) |  |  |  |  |
| Parent anxiety (11) |  |  |  |  |

1. **It is important for *all* graduating medical students to know how to perform basic pediatric otoscopy, defined as visualization of the tympanic membrane using an otoscope.**

- Strongly agree (1)
- Agree (2)
- Disagree (3)
- Strongly disagree (4)

1. **If it is not important for all students to learn this skill, are there particular residency-bound students that should learn this skill in medical school? Please check all that apply.**

- Pediatrics (1)
- Internal Medicine – Pediatrics (2)
- Family Medicine (3)
- Emergency Medicine (4)
- Otolaryngology (5)
- Other (6)

1. **If other, please specify:**

_________________________________________________________________________

1. **A curriculum regarding how to teach the pediatric ear exam to medical students in direct patient care settings would help me to be more effective in teaching this skill.**

- Strongly agree (1)
- Agree (2)
- Disagree (3)
- Strongly disagree (4)

The next set of questions relate to your own clinical experience with pediatric otoscopy.

1. **Which criteria on the physical exam do you use to diagnose the majority of your patients with acute otitis media? Check all that apply.**

- Moderate to severe bulging of the TM (1)
- Distinct erythema and poor mobility of the TM (2)
- Poor mobility and fluid levels of the TM (3)
- Retraction and abnormal color of the TM (4)
- Poor mobility of the TM (5)

1. **The AAP Guidelines for the diagnosis of Acute Otitis Media are helpful to me.**

- Strongly agree (1)
- Agree (2)
- Disagree (3)
- Strongly disagree (4)

1. **In which clinical situations do you use pneumatic otoscopy? Check all that apply.**

- To diagnose AOM (1)
- To diagnose OME (2)
- Only when I am unsure of the diagnosis (3)
- I do not use insufflation (4)

1. **I find it difficult to remove cerumen in the young child (age < 5 years)**

- Strongly agree (1)
- Agree (2)
- Disagree (3)
- Strongly disagree (4)

1. **Do you remove cerumen with direct visualization (using a curette directly through the speculum)?**

- Yes (1)
- No (2)

1. **Were you taught how to perform cerumen removal on a young child (age < 5 years)?**

- Yes (1)
- No (2)

1. **Where were you taught? Check all that apply.**

- Medical school pediatric clerkship (1)
- Medical school/non-pediatric clerkship (2)
- Residency (3)
- Post-residency (4)

1. **Of the children whose ears you have examined in the last three months, in approximately what percentage did you need to remove cerumen?**

- 0% (1)
- 1-20% (2)
- 21-49% (3)
- 50-74% (4)
- 75-99% (5)
- 100% (6)

1. **Were you taught how to perform pneumatic otoscopy on a young child (age < 5 years)?**

- Yes (1)
- No (2)

1. **Where were you taught? Check all that apply.**

- Medical school/pediatric clerkship (1)
- Medical school/non-pediatric clerkship (2)
- Residency (3)
- Post-residency (4)

1. **Skill in pneumatic otoscopy is important to the diagnosis of AOM**

- Strongly agree (1)
- Agree (2)
- Disagree (3)
- Strongly disagree (4)

The final questions relate to you as a provider

1. **How many years have you practiced ambulatory pediatrics (post-residency)?**

- 0-5 (1)
- 5-15 (2)
- 15-25 (3)
- 25+ (4)

1. **What year did you complete residency training in pediatrics?** _____________________
2. **How would you define your residency-training program?**

- Academic/tertiary care (1)
- Community pediatrics (2)

1. **How would you define your practice setting?**

- Urban/major city ( population > 350,000) (1)
- Suburban or mid-sized city (population 50,0000-350,000) (2)
- Small city ( population < 50,000) (3)

1. **In what type of practice do you work? Check all that apply.**

- Private practice (1)
- Academic center (2)
- Solo practitioner (3)
- Corporate-owned (4)
- FQHC of government-funded (5)
- Other (6)

1. **Please provide any other comments on the practice or the teaching of pediatric otoscopy.**

________________________________________________________________________________________________________________________________________________________________________________________________________________________________________________________________________________________________________________
